# Describing Self-Reported Penicillin Allergy Using a Penicillin Allergy Risk Tool (PEN-FAST) in an Outpatient Setting at a Tertiary Hospital in Saudi Arabia

**DOI:** 10.7759/cureus.51322

**Published:** 2023-12-30

**Authors:** Nour Baghdady, Hamzah N Alothmany

**Affiliations:** 1 Pharmacy Practice, King Abdulaziz University, Jeddah, SAU; 2 Pharmacy School, King Abdulaziz University, Jeddah, SAU

**Keywords:** prevalence, hypersensitivity, saudi arabia, pen-fast, penicillin allergy

## Abstract

Introduction

Penicillin is a widely used antibiotic and is frequently reported as a cause of allergic reactions. However, many individuals reporting penicillin allergies are later found to be tolerant. This study, conducted in an outpatient setting at King Abdulaziz University Hospital (KAUH) in Jeddah, Saudi Arabia, aimed to assess the prevalence of self-reported penicillin allergy and to evaluate further the risk of a positive penicillin allergy test using the PEN-FAST tool.

Methods

A cross-sectional study was conducted via in-person questionnaires with patients in the waiting area at the outpatient clinics of KAUH in Jeddah, Saudi Arabia.

Results

Among 140 participants, 4% reported a penicillin allergy, with most identifying their allergies based on symptoms. None of these allergies resulted in severe reactions. Notably, 50% reported nausea and itching as symptoms. The PEN-FAST tool categorized 33.3% as moderate risk and 50% as low to very low risk for a positive penicillin skin test. One participant was confirmed to be allergic via a skin prick test.

Discussion and conclusion

The prevalence of reported penicillin allergy was lower in our study than that previously reported. Evaluation of the PEN-FAST score demonstrated that this prevalence is even lower at 2%. While this single-center study offers valuable insights, further research in diverse healthcare settings is required to validate these findings and refine our understanding of penicillin allergies.

## Introduction

Penicillin is a beta-lactam antibiotic and is one of the most widely used antibiotics in the world [1]. Penicillin is frequently reported as a culprit in allergic reactions; however, upon further evaluation, most individuals are found not to be allergic. In the United States, 10% of patients report a penicillin allergy, but when further assessed, 90% of these are found to be tolerant of penicillin [2]. Similarly, a cross-sectional study conducted in Saudi Arabia assessed the prevalence and characteristics of self-reported penicillin allergy to be 9.5%. Upon further assessment, 11.4% were identified as not having a true penicillin allergy [3].

Patients believed to be allergic to penicillin are more likely to avoid other classes of antibiotics, such as cephalosporins, and are more likely to use broader-spectrum antibiotics [1]. This puts them at higher risk of side effects, increased hospital stay, and a higher risk for infection by *Clostridioides difficile* (C. diff), vancomycin-resistant *Enterococci* spp. (VRE), and methicillin-resistant *Staphylococcus aureus* (MRSA) [4-7].

Penicillin allergy testing is the current standard of care to confirm the presence, or lack thereof, of a true IgE-mediated allergy using the skin-prick test, drug challenge, or a combination of both [1,8]. However, these strategies require a specialist’s interpretation and are labor intensive. Recently, the PEN-FAST penicillin allergy risk tool has been validated to evaluate individuals reporting a penicillin allergy for their risk of testing positive on a skin prick test. This tool works by using information from a patient's history to stratify their risk for an allergy. It does so by assessing three clinical criteria: time since the last episode, phenotype of the reaction, and whether treatment was used. This is done through a set of four questions [9]. The answer for each question is given a score as laid out in Table 5 (given in the Appendices section of this article). The risk for a positive skin test can be predicted by the total score on these questions depicted in Table 6 (Appendices section) [9]. This tool has demonstrated a high negative predictive value of 96.3% in those scoring < 3 [9]. It has many advantages including its ease of use, being non-invasive, validated for use at point of care, and is now available as an online tool that can be used free of charge [9].

King Abdulaziz University Hospital (KAUH) in Jeddah is a major tertiary hospital that serves thousands of patients a year. The outpatient clinics care for over 200 thousand patients annually. The aim of this study is to describe the frequency of self-reported penicillin allergy in patients visiting an outpatient clinic at KAUH and to evaluate the risk for a positive penicillin allergy test utilizing the PEN-FAST tool in those who report an allergy.

## Materials and methods

Study design

We conducted a cross-sectional study through in-person questionnaires with patients visiting the outpatient clinics at KAUH in Jeddah, Saudi Arabia in the month of March 2023. Investigators asked patients visiting the outpatient clinic about their interest in participating in a questionnaire regarding penicillin allergies. They were informed what the questionnaire was assessing and that their participation, if accepted, would be voluntary, confidential, and would not affect their care at KAUH. Participants filled out a questionnaire that was built through a University-hosted Google Form. All responses were registered by the researchers.

Questionnaire

The questionnaire was built in Arabic and English languages and encompassed three main sections: baseline characteristics of participants, questions related to penicillin allergy, and questions necessary for calculating the PEN-FAST score. To develop the questionnaire, a literature review was conducted to identify pertinent questions.

The first two sections of the questionnaire were validated internally by having an independent allergist evaluate the questions and test-run them. The third section, (the PEN-FAST questions), was globally validated previously and has already been used [9]. The questionnaire was piloted prior to the study to identify any redundancies and confusion. To see the detailed questions, please refer to Tables 5-7 (Appendix A, B, and C).

Sample size

The outpatient clinic serves on average 200,000 patients a year. Previous studies conducted in Saudi Arabia and the USA found that the prevalence of penicillin allergy ranged from 9% to 11.5% [3,10]. Based on the results of these two studies, we estimated that around 10% of respondents were expected to report a penicillin allergy. With a 95% confidence interval and a margin of error of 5%, the calculated sample size was determined to be 139. Rounding to the nearest 10, the required sample size was determined to be 140.

Inclusion criteria

Participants were interviewed and included in our analysis if they were in the waiting area of the outpatient clinics at KAUH in March 2023, were 18 years of age or older, spoke either Arabic or English, and consented to participate.

Outcomes

The primary outcome was to determine the prevalence of self-reported penicillin allergy at our institution. The secondary outcome was the calculated PEN-FAST score for those reporting a penicillin allergy.

Statistical analysis

We used descriptive statistics to summarize our data, including medians with interquartile ranges (IQR) and percentages.

Ethics

This study was reviewed and approved by the Research Ethics Committee at King Abdulaziz University Faculty of Medicine (approval number: HA-02-J-008).

## Results

Participant demographics

A total of 140 respondents were interviewed and included in the analysis. The age distribution of participants varied, with the youngest being 18 years of age and the oldest 75 years. Participants were generally highly educated, with more than 85% holding a high school degree or higher. Most participants were Saudi citizens (70%) (Table 1).

**Table 1:** Baseline characteristics

Baseline Characteristics	N=140
Age (years); Median (IQR)	42 (30-53)
Gender: Female, n (%)	70 (50%)
Education status, n (%)	
No education	4 (2.9%)
Elementary	8 (5.7%)
Middle school	8 (5.7%)
High school	44 (31.4%)
Bachelor’s degree	54 (38.6%)
Postgraduate	12 (8.6%)
Diploma	10 (7.1%)
Nationality; Saudi, n (%)	99 (70.1%)
Comorbidities	
Asthma	8 (5.8%)
Cardiac Conditions	17 (12.2%)
Diabetes	24 (17.3%)
Hypertension	33 (23.7%)
None	72 (51.8%)
Other	26 (18.7%)
Report a Penicillin Allergy, n (%)	6 (4.3%)

Prevalence and characteristics of penicillin allergy

Out of the 140 participants, six (4%) reported a penicillin allergy. Table 2 describes the baseline characteristics of respondents reporting an allergy. In four (66.7%) cases, participants identified their allergies based on signs and symptoms they experienced. Only one individual described undergoing a penicillin skin test. None of the reported allergies resulted in severe reactions leading to hospitalization, nor have any reported experiencing anaphylaxis, angioedema, or severe cutaneous reactions. Notably, 50% of the participants reported nausea and pruritus/itching as symptoms. One participant reported concomitant use of a non-steroidal anti-inflammatory drug (NSAID), and another reported the use of an angiotensin-converting enzyme inhibitor (ACEI) at the time penicillin was used. Only two (33.3%) participants reported treatment with an oral antihistamine. A comprehensive overview of the characteristics of reported penicillin allergy reactions is provided in Table 3.

**Table 2:** Baseline characteristics of those who reported a penicillin allergy

Baseline characteristics	N=6
Age (years); Median (IQR)	43.5 (10)
Gender: Female, n (%)	3 (50%)
Education status > high school, n (%)	6 (100%)
Nationality; Saudi, n (%)	6 (100%)
Comorbidities	
Asthma	1 (16.7%)
Cardiac Conditions	1 (16.7%)
Diabetes	1 (16.7%)
Hypertension	33 (23.7%)
Other (hypothyroidism)	1 (16.7%)
None	3 (50%)

**Table 3:** Penicillin allergy characteristics

Characteristics	N=6
How do you know you are allergic to penicillin?	
Experienced signs and symptoms	4 (66.7%)
Confirmed by a skin test	1 (16.7%)
Told by parents	1 (16.7%)
When did you last experience a reaction?	
Five years or less	3 (50%)
More than five years	3 (50%)
What medication triggered your reaction?	
Penicillin	1 (16.7%)
Amoxicillin	1 (16.7%)
Amoxicillin-Clavulanate (Augmentin)	3 (50%)
Don’t remember	1 (16.7%)
How soon did the reaction occur after taking the medication?	
Less than 1 hour	4 (66.7%)
1 hour to 72 hours	2 (33.3%)
Beyond 72 hours	0
What happened during the reaction?	
Skin: red, raised, itchy bumps	2 (33.3%)
Skin: Pruritus, itching	3 (50%)
Decreased blood pressure	1 (16.7%)
Nausea	3 (50%)
Abdominal pain	1 (16.7%)
Dyspnea	1 (16.7%)
Swelling of the face	1 (16.7%)
Fatigue	1 (16.7%)
Have you experienced or were told to have anaphylaxis or angioedema?	
No	6 (100%)
Yes	0
Did you experience severe cutaneous reactions?	
No	6 (100%)
Yes	0
Did you need treatment for your allergic reaction?	
Yes (antihistamines)	2 (33.3%)
No	4 (66.7%)
At the time of the reaction. were you on any of the following classes of medications?	
Antibiotics	1 (16.7%)
NSAIDs	1 (16.7%)
ACEI	1 (16.7%)
No	3 (50%)
Are you allergic to any other antibiotic?	
Yes	1 (16.7%)
No	5 (83.6%)
Are you allergic to any other medication?	
Yes (Ibuprofen)	1 (16.7%)
No	5 (83.6%)

PEN-FAST scores

According to the calculated PEN-FAST scores, 2 (33.3%) participants were determined to be at moderate risk for a positive penicillin skin test and 3 (50%) to be of low risk to very low risk (Table 4). In one case, a PEN-FAST score could not be calculated since they had a confirmed positive penicillin allergy skin test.

**Table 4:** PEN-FAST scores and characteristics of each participant PEN-FAST: Penicillin Allergy Risk Tool

	PEN-FAST Scores	Gender	Age	Comorbidities
Participant 1	2	Female	43	Hypothyroidism
Participant 2	3	Male	56	Asthma, Diabetes, and hypertension
Participant 3	3	Female	29	No diseases
Participant 4	0	Female	53	Hypertension
Participant 5	NA (confirmed by skin test)	Male	40	No diseases
Participant 6	2	Male	44	No diseases

## Discussion

Being labeled with penicillin-allergy comes with its own consequences, ranging from an increased risk of multidrug-resistant infections such as methicillin-resistant *Staphylococcus aureus* (MRSA) or vancomycin-resistant *Enterococcus* (VRE), increased length of hospital stay, hospital admissions and outpatient visits [4,7,11,12]. Very limited data exists on reported penicillin allergy rates in Saudi Arabia. None of which further evaluated it using validated tests, such as skin tests or drug challenges, or validated point of care tools, such as the PEN-FAST tool. Studies have shown that when a penicillin allergy test/procedure is done effectively it leads to an increase in the usage of a beta-lactam antibiotic, while also being cost-effective [12-16].

This on-site cross-sectional study evaluated the prevalence of reported penicillin allergy in the outpatient clinics at a tertiary hospital in Jeddah and used the PEN-FAST tool for further evaluation. Our study found that the prevalence of reported penicillin allergy was 4%. Upon further evaluation with the PEN-FAST tool, only two were identified to be at moderate risk of a true penicillin allergy. They achieved this score due to requiring treatment by antihistamine, and their most recent reaction to the penicillin allergy was less than five years ago. We were unable to assess if the experienced symptoms (such as face swelling) were due to the receipt of penicillin or other medications such as NSAID. One reported a confirmed penicillin allergy determined by a skin prick test. Taken together, this indicates that of the total population interviewed, 2% of our study population were at true risk for a penicillin allergy. The remaining three participants reporting an allergy were found to be low to very low risk. This puts them at risk of developing the consequences mentioned above. This further highlights the ease of use and implementation of the use of the PEN-FAST tool as it is less invasive and can be done at the point of care [9].

The reported penicillin allergy rate in our study was lower than that previously reported in Saudi Arabia, 9.5%, and other countries [1,3,10,16]. The higher prevalence reported in the other study conducted in Saudi Arabia could be due to several factors [3]. First, it was a cross-sectional study conducted using online surveys that were more widely distributed nationally, including respondents from different regions of Saudi Arabia via social media platforms such as WhatsApp. This online distribution could have favored responses from respondents who are more fluent with technology. Second, those reporting an allergy might be more willing to respond to such questionnaires, putting this study at risk for selection bias [3]. Finally, most respondents in this study were female (76%); being female is an independent risk factor for a penicillin allergy and could have led to a higher reported rate [3,17,18]. 

The strength of our study lies in it being conducted in person, which allowed for the inclusion of those with limited technology experience and prevented duplication in responses. Also, we utilized the PEN-FAST score to further evaluate the risk for a positive penicillin allergy test, which was not done in other studies conducted in Saudi Arabia. A score less than 3 has a high negative predictive value (NPV) of 96.3% [9]. This gave us a deeper understanding of those reporting an allergy.

Our study is limited in it being single centered. This can affect the generalizability of our results to other institutions or regions in Saudi Arabia. Also, our study was conducted in the outpatient setting, these results cannot be generalizable to the inpatient setting. Additionally, the study is at risk of recall bias. Participants may have omitted or changed important details related to their penicillin reaction due to difficulty in recalling what exactly happened. Finally, those deemed to be at moderate risk for a penicillin allergy were not further evaluated using a drug challenge or a skin test. Future studies, including the next steps for those found to be at higher risk for an allergy, are warranted. Ideally, such studies should be larger, multi-centered, representing different regions of the country, and should explore the effects of being labeled with an allergy.

## Conclusions

In summary, our study reveals a lower prevalence of self-reported penicillin allergies in an outpatient setting. It highlights the importance of accurate allergy assessment using tools like the PEN-FAST score, as a significant portion of those reporting a penicillin allergy were found to be at low or very low risk. Addressing this issue is crucial for optimizing patient care, reducing healthcare costs, and preserving the effectiveness of antibiotics. Further research in diverse healthcare settings is needed to validate our findings and refine our understanding of penicillin allergies.

## Appendices

Appendix A:  PEN-FAST allergy risk tool

**Table 5:** PEN-FAST score interpretation Trubiano JA, Vogrin S, Chua KYL, et al.: Development and validation of a penicillin allergy clinical decision rule. JAMA Intern Med. 2020, 180:745-752. 10.1001/jamainternmed.2020.0403 Permission was obtained from the original publishers to repurpose this table.

Total Score	Risk for a Positive Penicillin Allergy Test
0	Very low (<1%)
1-2	Low (5%)
3	Moderate (20%)
> 4	High (50%)

Appendix B: PEN-FAST score interpretation

**Table 6:** PEN-FAST Allergy Risk Tool Trubiano JA, Vogrin S, Chua KYL, et al.: Development and validation of a penicillin allergy clinical decision rule. JAMA Intern Med. 2020, 180:745-752. 10.1001/jamainternmed.2020.0403 Permission was obtained from the original publishers to repurpose this table.

Questions- Yes/No	Score
PEN - Penicillin allergy reported by patient	0
F - Five years or less since reaction	2
A - Anaphylaxis or angioedema	2
S - Severe cutaneous adverse reaction	2
T - Treatment required for reaction	1

Appendix C: Questionnaire* *

**Table 7:** Full questionnaire The first two sections were validated by an independent allergist. The third section (PEN-FAST) was already globally validated and can be found in the following citation: Trubiano JA, Vogrin S, Chua KYL, et al.: Development and validation of a penicillin allergy clinical decision rule. JAMA Intern Med. 2020, 180:745-752. 10.1001/jamainternmed.2020.0403

Full questionnaire:	
We are a pharmacy research team that is interested in exploring penicillin allergies in our hospital through a questionnaire. This questionnaire can take about 5-10 minutes. Your participation would be greatly appreciated and is completely voluntary and will not affect your care at KAUH. If at any point you would like to stop or withdraw, you can do so with no repercussions. Your identity will remain confidential. Would you like to participate? Yes (move to next section) No (close the questionnaire)	
First section (baseline characteristics):	
Age:	_____
Sex:	Male, Female
Education background:	Elementary, Middle school, High school, College graduate, Diploma, Postgraduate
Citizenship status:	Saudi citizen, Non-Saudi
Comorbidities (check all that apply):	Asthma, Cardiac conditions, Eczema, Diabetes, Hypertension, Others:_____________, None
Section 2 (Penicillin allergy questions):	
PEN- Are you allergic to penicillin?	Yes (continue on with the questionnaire) No (close the questionnaire)
How do you know?	Informed by parent, Informed by physician, Experienced S&S, Confirmed by allergy skin test, Drug challenge, Don’t remember
Which medication triggered your reaction? (check all that apply)	Penicillin ,Amoxicillin, Amoxicillin-clavulanate (Augmentin), Piperacillin-tazobactam, Other:____________ ,Don’t remember
How soon did the reaction occur after taking the medication?	< 1 hour, 1-72 hours ,72 hours – < 2 weeks ,2-6 weeks, Don’t remember
What happened? (Check all that apply)	Skin: red, raised, itchy bumps, Skin: flushing, Skin: pruritus, itching Skin: Rash Skin: blistering Skin: Peeling Skin: sloughing Fever Swelling: eyes, face, lips, tongue Circulatory: Decreased blood pressure GI: nausea GI: vomiting GI: abdominal pain GI: abdominal cramps Respiratory: Dyspnea Respiratory: Wheeze-bronchospasm Respiratory: Hypoxemia Other:_____________ Don’t remember
Are you allergic to any other antibiotics?	Yes Which ones?__________________ No Don’t know
Are you allergic to any other medications?	Yes Which ones?__________________ No Don’t know
Section 3: PEN-FAST questions:	
F- When did you last experience a reaction?	= or < than 5 years ago (scores 2 points) , > 5 years ago, Don’t remember
A – Were you told to have experienced anaphylaxis or angioedema?	Yes (scores 2 points) , No, Don’t remember
S – Did you experience a severe cutaneous adverse reaction?	Yes (scores 2 points), No, Don’t remember
T – Did you need to treat your allergic reaction?	Yes (scores 1 point) What treatment?__________________ No Don’t remember
